# A discovery study of daunorubicin induced cardiotoxicity in a sample of acute myeloid leukemia patients prioritizes P450 oxidoreductase polymorphisms as a potential risk factor

**DOI:** 10.3389/fgene.2013.00231

**Published:** 2013-11-11

**Authors:** Joanna M. Lubieniecka, Jinko Graham, Daniel Heffner, Randy Mottus, Ronald Reid, Donna Hogge, Tom A. Grigliatti, Wayne K. Riggs

**Affiliations:** ^1^Department of Zoology, Life Sciences Institute, University of British ColumbiaVancouver, BC, Canada; ^2^Department of Statistics and Actuarial Science, Simon Fraser UniversityBurnaby, BC, Canada; ^3^Department of Medicine, University of British ColumbiaVancouver, BC, Canada; ^4^Faculty of Pharmaceutical Sciences, University of British ColumbiaVancouver, BC, Canada; ^5^Terry Fox Laboratory, British Columbia Cancer AgencyVancouver, BC, Canada

**Keywords:** anthracycline induced cardiotoxicity, daunorubicin, acute myeloid leukemia, P450 oxidoreductase variants, complex trait, global test, adverse drug reactions

## Abstract

Anthracyclines are very effective chemotherapeutic agents; however, their use is hampered by the treatment-induced cardiotoxicity. Genetic variants that help define patient's sensitivity to anthracyclines will greatly improve the design of optimal chemotherapeutic regimens. However, identification of such variants is hampered by the lack of analytical approaches that address the complex, multi-genic character of anthracycline induced cardiotoxicity (AIC). Here, using a multi-SNP based approach, we examined 60 genes coding for proteins involved in drug metabolism and efflux and identified the *P450 oxidoreductase* (*POR*) gene to be most strongly associated with daunorubicin induced cardiotoxicity in a population of acute myeloid leukemia (AML) patients (FDR adjusted *p*-value of 0.15). In this sample of cancer patients, variation in the *POR* gene is estimated to account for some 11.6% of the variability in the drop of left ventricular ejection fraction (LVEF) after daunorubicin treatment, compared to the estimated 13.2% accounted for by the cumulative dose and ethnicity. In *post-ho*c analysis, this association was driven by 3 SNPs—the rs2868177, rs13240755, and rs4732513—through their linear interaction with cumulative daunorubicin dose. The unadjusted odds ratios (ORs) and confidence intervals (CIs) for rs2868177 and rs13240755 were estimated to be 1.89 (95% CI: 0.7435–4.819; *p* = 0.1756) and 3.18 (95% CI: 1.223–8.27; *p* = 0.01376), respectively. Although the contribution of *POR* variants is expected to be overestimated due to the multiple testing performed in this small pilot study, given that cumulative anthracycline dose is virtually the only factor used clinically to predict the risk of cardiotoxicity, the contribution that genetic analyses of *POR* can make to the assessment of this risk is worthy of follow up in future investigations.

## Introduction

Anthracyclines are very effective chemotherapeutic agents used in the treatment of a wide variety of leukemias, lymphomas, and solid tumors. Due to their high efficacy and broad-spectrum application, these agents are being used despite the associated side effects. The most adverse side effect of anthracycline therapy is cardiotoxicity, which often leads to chronic cardiomyopathy and in some cases congestive heart failure and death (Sawyer et al., 2010). In addition to limiting the dose of the drug administered, several other strategies, including liposomal encapsulation, modification of anthracycline structure, and concurrent administration of iron chelators, have been used as attempts to lessen the cardiotoxic effect of anthracycline treatment (Sawyer et al., 2010). Despite these efforts, chronic cardiomyopathy remains as a life-long threat with the risk for chronic heart failure ranging from 3 to 26% depending on the dose of the drug (Yeh and Bickford, 2009).

The proposed risk factors for anthracycline induced cardiotoxicity (AIC) include cumulative dose, age, gender, pre-existent medical conditions, and combination therapy (Menna et al., 2012). Among these risk factors, the strongest is the cumulative dose of the drug. Consequently, physicians and regulators have set limits on the maximum anthracycline dose that can be used. However, the variability in sensitivity to anthracyclines is quite broad and some patients experience cardiotoxicity with doses as low as 200 mg/m^2^, while others tolerate doses as high as 1000 mg/m^2^ (Yeh and Bickford, 2009). These observations suggest that there is no truly “safe” anthracycline dose and bio-markers are needed to identify patients vulnerable to AIC, as well as those that can tolerate much higher doses of the drugs with little risk, and thus could benefit from more aggressive treatment (Estey, 2012; Menna et al., 2012).

The range of variability among patients in their sensitivity to anthracyclines cannot be fully explained by the known risk factors or pharmacokinetic parameters, and suggests there is an indispensible genetic component (Duan et al., 2007). Genetic contribution to the variability in sensitivity is further supported by the relatively high heritability of anthracycline cytotoxicity (*h*^2^ = 0.18–0.63) (Duan et al., 2007; Peters et al., 2011). A number of predisposing genetic variants have been reported; however, these do not explain all of the variability in risk (Wojnowski et al., 2005; Blanco et al., 2008, 2012; Rajic et al., 2009; Visscher et al., 2012). The limited success of genetic studies searching for variants that would explain all of the observed variability in AIC suggests that AIC is a complex phenotype with many genes, environmental factors, and gene-environment interactions contributing to its development (Duan et al., 2007). For such complex traits many genes (even hundreds) contribute to the final phenotype and variants at each of the loci will generally have a modest effect. Therefore, they are difficult to identify, and will often be missed by the standard single-variant based association tests (De Bakker et al., 2005; Kraft and Hunter, 2009; De Los Campos et al., 2010; Yang et al., 2010; Stringer et al., 2011). Moreover, single-variant based association tests ignore the possibility of synergistic and gene-environment interactions, which often results in missing potentially important loci (Ober and Vercelli, 2011; Wheeler et al., 2012). Increasing the size of the study sample will increase the power to detect variants that have modest effects. However, even a large (e.g., 10,000 subjects) study sample will have low power to detect any specific marker with the least stringent significance threshold (*p* = 10^−7^) in a genome-wide association study of thousands of SNPs (Kraft and Hunter, 2009). Therefore, in realistically sized study samples, the search for genetic variants of complex, polygenic traits requires a multi-variant based approach that allows for potential gene-environment interactions to identify genetic contributors of small or modest effect (De Bakker et al., 2005; Ober and Vercelli, 2011; Wheeler et al., 2012).

The exact causes of AIC are unclear; however, data accumulated to date indicates that anthracycline metabolism, either through generation of free radicals or toxic metabolites, or a combination of both, is a significant contributor to the drugs' cardiotoxicity (Sawyer et al., 2010; Menna et al., 2012). Therefore, we selected 60 genes coding for proteins involved in drug metabolism and efflux as candidates for a pilot project focused on identifying candidate markers of AIC in a sample of acute myeloid leukemia (AML) patients undergoing daunorubicin (DAUN) based treatment. Using a multi-variant based approach that allows for potential gene-anthracycline dose interactions, we identify genetic variants in the *POR* gene as potential markers of AIC among AML patients undergoing DAUN treatment.

## Materials and methods

### Patients and clinical outcome definition

Peripheral blood samples were obtained from 286 AML patients after informed consent and with approval from the Clinical Research Ethics Board of the University of British Columbia. The patient samples were obtained over an 8–10 years period and the samples and patient data were examined retrospectively. All patients received DAUN or mitoxantrone (MITOX) in combination with cytarabine for initial remission induction and subsequent consolidation therapy. For each patient cardiac function was monitored by left ventricular ejection fraction (LVEF) measurements determined with radionuclide ventriculogram (RVG) scans and/or echocardiograms. This patient population has been described in more detail elsewhere (Kim et al., 2012).

### Patient selection

The aim of this study was to identify genetic variants that render some patients vulnerable to the cardiotoxic side effect of anthracycline treatment. Since objective measurement of cardiotoxicity due to treatment (and not other causes) can only be done if cardiac function is assessed before and after treatment, only patients that had their LVEF measurements done before and after (less than a year after last dose of anthracycline) anthracycline treatment were used in subsequent statistical analyses (114 patients).

Percentage drop in LVEF was used as a measure of decrease in cardiac function after treatment. LVEF is a measure commonly used to monitor cardiac function in patients undergoing anthracycline treatment and allows for analysis of cardiotoxicity as a quantitative trait (Ewer and Ewer, 2009). A genetic statistical analysis of such traits is typically more powerful than an analysis of binary traits because it allows for discernment of differences in unaffected individuals. In addition, this approach eliminates the often imprecise process of binning individuals into subjective categories (such as cases and controls) (De Los Campos et al., 2010; Stringer et al., 2011).

Furthermore, to keep the treatment uniform across the study sample, patients undergoing MITOX based therapy were also removed, leaving 91 patients for the statistical analyses. A detailed description of this sample population is presented in Table 1.

**Table 1:** **Characteristics of the AML study sample**.

**Number of patients**		**Females 43**	**Males 48**	**Total 91**
Age	Average	48.2	48.7	48.4
	Range	19–74	20–72	19–74
DAUN cumulative dose [mg/m^2^]	Average	293.1	260.4	275.9
	Range	50–630	60–540	50–630

### Genotyping

DNA samples from all patients were genotyped for haplotype tagging (ht) and non-synonymous (ns) SNPs in 35 cytochrome p450s (CYPs), 23 oxidoreductases (reductases), and two ABC transporter genes (ABCB1 and ABCG2). The genes were selected based on their documented or possible role in anthracycline metabolism (reductases) or efflux (ABC transporter genes). The CYP genes were also added due to their involvement in redox cycling, which is also implicated in AIC. The gene sequence used for SNP selection included 5 kb of gene flanking region upstream from the start codon and 10kb downstream from the stop codon. The htSNPs were selected from the HapMap database, release #28 (http://hapmap.ncbi.nlm.nih.gov/) using the Tagger tool and the following cut-offs: linkage disequilibrium (LD) value (*r*^2^) = 0.8 and the minor allele frequency (MAF) cutoff = 1% (De Bakker et al., 2005). Using these criteria a total of 921 SNPs were selected to be genotyped. Genotyping was performed using the Sequenom genotyping system (Sequenom Inc., San Diego, CA). For quality control, SNPs with call rate of less than 95%, those with minor allele frequencies less than 1% or not in Hardy-Weinberg equilibrium (*p* > 0.001) were removed from the raw dataset, leaving a total of 465 SNPs for statistical analysis. All 91 patients had call rates of at least 90% across the 465 SNPs.

### Statistical analysis

All statistical analyses were performed in the R programming environment (R-Development-Core-Team, 2011). Genotypes were coded as 0, 1, and 2 according to the number of copies or “dosage” of the minor allele. Sporadically missing genotypes were imputed with the posterior expected dosage of the minor allele, as estimated by *BEAGLE v3.3.1* (Browning and Browning, 2009).

As expected, the distribution of percent drop in LVEF was highly skewed, with most patients experiencing no or very little change but a few experiencing drops over 10%. To stabilize variance and ensure validity of permutation tests, the percent drop in LVEF was transformed to the logarithmic scale (base 2), after shifting up values by 1.

Potential confounding variables included age, gender, and cumulative DAUN dose. An additional source of potential confounding is population stratification due to ethnic differences. In this study ethnicity was measured by the top three principal components (PCs) of genetic variation (Price et al., 2006). To capture ethnicity as completely as possible, additional genotype data for 195 SNPs in 27 oxidative stress response genes (generated for the patients through a separate study) were added to the genotype set used to define the PCs. To reduce the number of potential confounding variables, forward stepwise selection of these variables in a linear regression model of transformed LVEF drop was applied, based on predictive ability as measured by Akaike's information criterion (Venables and Ripley, 2002). Subsequent genetic analyses were adjusted for the predictive variables only. This base model was used to estimate the proportion of variability in the transformed LVEF drop explained by the potential confounding variables, so that it could be compared to the proportion of variability explained by SNPs in the set of significant candidate genes. Candidate genes explaining a proportion of the variability in LVEF drop that is comparable to variables such as dose or ethnicity that are suspected to influence cardiotoxicity are compelling candidates for further investigation.

To test for association of each SNP, a linear model was fitted to the log-transformed LVEF percent drop that adjusts for dose, the first two genetic PCs, the SNP additive “main effect” and the SNP's linear interaction with dose. To summarize the joint significance of the SNP main effect and interaction, a likelihood ratio statistic was calculated. The exact *p*-value for this test statistic was approximated by 2000 permutation replicates. To control the expected proportion of false discoveries among rejected hypotheses (the false discovery rate or FDR), the nominal *p-values* were corrected for multiple testing of SNPs (Benjamini and Hochberg, 1995). The Manhattan plot of the nominal *p*-values was done using the *Haploview* program (Barrett, 2009).

Testing genes rather than individual SNPs for association with cardiotoxicity reduces the multiple-testing penalty, enhancing power in this pilot study. Therefore, each of the 60 candidate metabolic genes was tested for association with cardiotoxicity using a global test of its SNP effects and their linear interactions with dose, as implemented in the Bioconductor package *globaltest* (Goeman et al., 2006). This test was chosen because comparative simulation studies of gene-based tests demonstrate that it is well-powered under a variety of alternative hypotheses (Pan, 2009). The global test is motivated by a linear mixed model which adjusts for the fixed effects of confounding variables but specifies random effects for SNPs and their interactions with dose. To control the FDR, the nominal *p*-values were corrected for multiple testing of genes (Benjamini and Hochberg, 1995). All *p*-values for gene-based associations were obtained from permutation tests based on 200,000 permutation replicates.

In *post-hoc* analyses, gene-based associations significant at an FDR level of 20% were further examined in order to understand which SNPs within a gene were driving the association. Specifically, the global test statistics were decomposed into the contributions from each SNP main effect and interaction with dose in the mixed model, using the inheritance procedure as implemented in the *covariates()* function of the *globaltest* package (Goeman and Finos, 2012). For SNPs within a gene, a standard level of 5% was applied to control the family-wise error rate (FWER) and to minimize the number of false-positive SNPs or SNP-by-dose interactions incorrectly identified as drivers of the gene-based association. When decomposing a gene-based association, SNPs were identified as important if they were within an associated gene (at FDR level 20%) and were also significant within the gene (at FWER level 5% in the *post-hoc* analysis), either through their main effect or through their interaction with dose.

To estimate the proportion of variability in LVEF drop explained by the candidate metabolic genes, important SNPs in associated genes were included in an ordinary (fixed effects) regression model with log-transformed LVEF values as the response and terms for dose, ethnicity, SNPs and linear interactions of the SNPs with dose. The proportion of variability in the log-transformed LVEF drop explained by the SNPs in candidate genes was then estimated from the fitted model, without adjusting for multiple testing.

Pairwise LD between significant SNPs and their haplotype phase were estimated using the PLINK package (Purcell et al., 2007).

## Results

Initially 286 patients were genotyped in this blinded, retrospective study. However, subsequent analysis of clinical data identified variability in assessment of cardiac function. Since the outcome under investigation was the cardiotoxic effect of anthracycline treatment and not general cardiac health of the patients, only patients with LVEF measurements done before and after treatment were included in statistical analyses (114 patients). From this sample only those patients treated with the anthracycline DAUN were retained for the statistical analysis (91 patients). Although this patient selection reduced the sample size, it had the benefit of making the sample much more uniform and therefore less prone to bias from hidden confounding.

To reduce the dimensionality of the data set, we first determined if the known potential confounding variables (DAUN dose, patient age, gender, and ethnicity) contributed to the variance in LVEF drop. Since information on ethnicity was not available we applied PC analysis using 660 SNPs in 89 genes to determine the three major axes of genetic variation. These axes were taken as a summary of the underlying large-scale population structure and used to correct for population stratification due to ethnic differences. Forward selection of the potential confounding variables found that anthracycline cumulative dose and the top two genetic PCs are predictive of LVEF drop. The potential confounding factors, age, and gender, as well as the third genetic PC were not predictive of LVEF drop and were therefore excluded from further analyses. Anthracycline cumulative dose and the top two genetic PCs accounted for an estimated 13.2% of the variation in the log-transformed LVEF drop.

**Figure 1:**
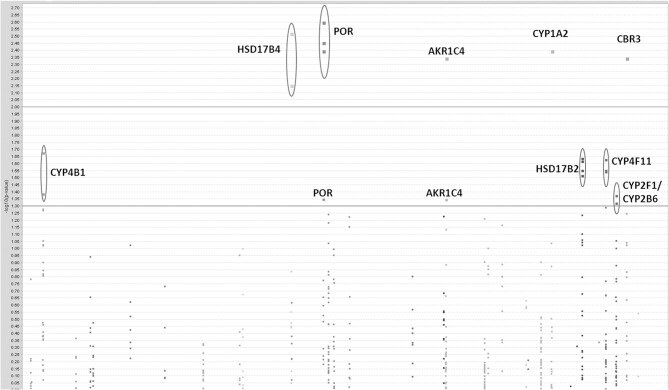
**Results of single-SNP based tests of association with drop in LVEF**. A Manhattan plot of log transformed nominal *p*-values against SNP chromosomal localization.

Initially we considered 921 SNPs in 60 metabolic and efflux genes for their effect on % drop in LVEF (Supplemental Table 1). After eliminating SNPs that did not pass QC filters (see Materials and Methods), a total of 465 SNPs remained for tests of association. Tests for the effect of individual SNPs on drop in LVEF identified 25 SNPs at a nominal *p*-value of = 0.05 (Table 2). As could be expected, given the small number of patients relative to the number of genetic variants, none of these SNPs survived a correction for multiple comparisons at FDR level of 20%. This suggests that if any of the analyzed SNPs has an effect on LVEF individually, it is probably too small to be detected in the small sample of patients. However, a Manhattan plot of the nominal *p*-values for the single SNP association tests (Figure 1) suggests that some genes are “enriched” for SNPs with low *p*-values. This was especially true for the *POR* and *HSD17B2*, where more than three SNPs in each gene were found to be associated with LVEF drop at a nominal *p*-value of less than 0.05. Some of these SNPs, such as the four SNPs in *HSD17B2*, were found to be in strong LD (*r*^2^ > 0.8) explaining their concurrent association with LVEF drop (Table 2). However, others were in weak LD (<0.6) suggesting that their concurrent association with the outcome is due to factors other than linkage and flags the gene as an interesting locus.

**Table 2:**
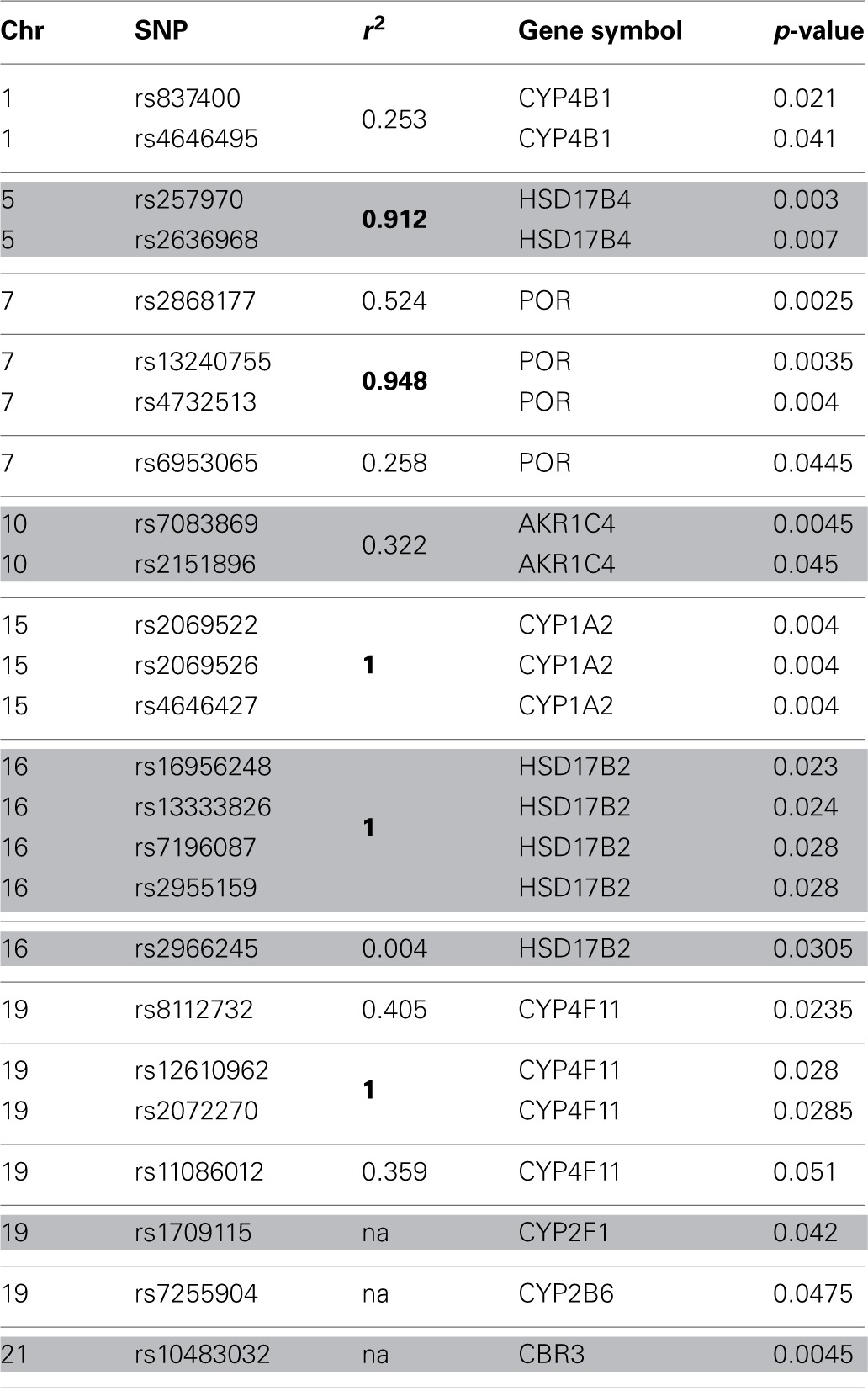
**Top 25 SNP identified through single SNP tests of association**.

The p-values have not been corrected for multiple comparisons. r^*2*^—squared correlation coefficient, a measure of linkage disequilibrium (LD). For genes with more than 2 SNPs the r^2^ value refers to LD between the SNP and a reference SNP. r^2^ > 0.8.

**Figure 2:**
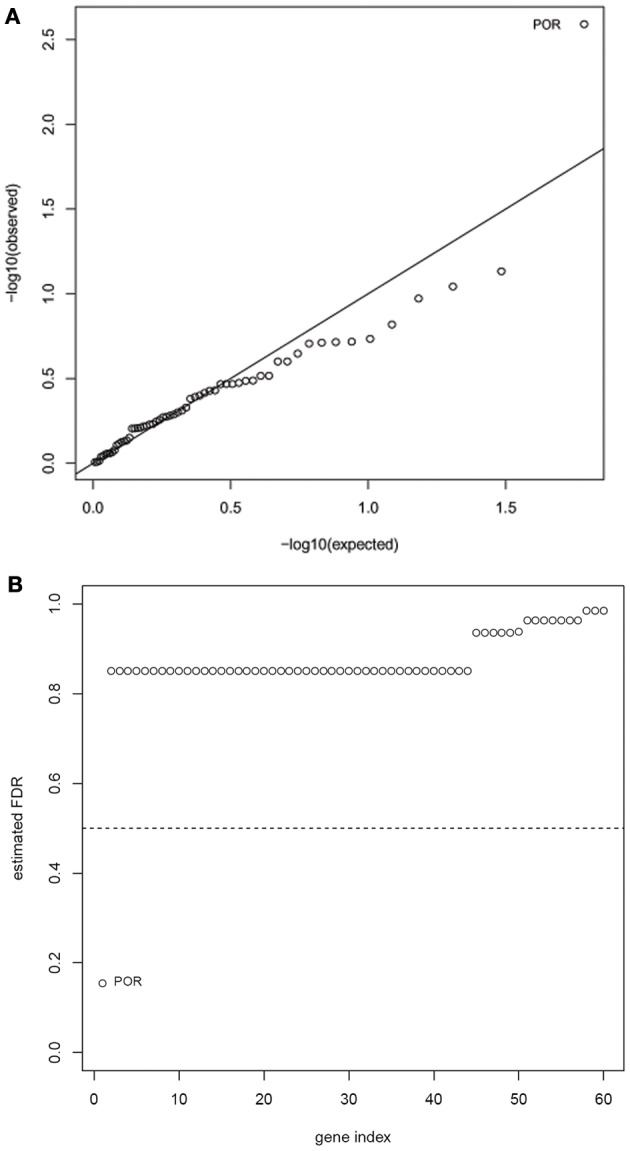
**Results of gene-based tests of association with drop in LVEF. (A)** Quantile-quantile plot of nominal *p*-values for the 60 genes included in analysis—the observed *p*-values are plotted against *p*-values expected under the null hypothesis.**(B)** Benjamini-Hochberg false-discovery rates (FDRs) for the 60 analyzed genes. The strongest signal was detected for the POR gene (FDR adjusted *p*-value = 0.15).

Another limitation of the Manhattan plot is that the graphic downplays the gene size, as well as the differences in the number of SNPs between genes. Therefore, we applied a multi-SNP based approach in which each of the 60 genes was tested for association with drop in LVEF using a global test of its SNPs and their linear interactions with dose (Goeman et al., 2006). This test statistic adjusts for gene size, number of SNPs, and LD between the SNPs. The unadjusted *p*-values for the 60 genes are shown in the quantile-quantile plot (Figure 2A), in which observed *p*-values are plotted against *p*-values expected under the null hypothesis (no genetic association). The FDR-adjusted *p*-values for the 60 genes are displayed in Figure 2B, with the strongest signal detected for the *POR* gene (FDR adjusted *p*-value = 0.15). After accounting for the effects of anthracycline dose and ethnicity, the proportion of variability in the LVEF drop attributable to POR is estimated to be 11.6% in *post-hoc* analyses.

Next, the overall association of LVEF drop with *POR* was decomposed into the contributions of each SNP main effect and its interaction with dose. Three of the 13 SNPs in *POR*—rs2868177, rs13240755, and rs4732513—were significant drivers of the association at 5% FWER level, through their linear interaction with cumulative DAUN dose. Given that the SNPs rs13240755 and rs4732513 are in strong LD (*r*^2^ = 0.948; D′ = 1.000) only rs13240755 and rs2868177 were included in further analyses.

**Figure 3:**
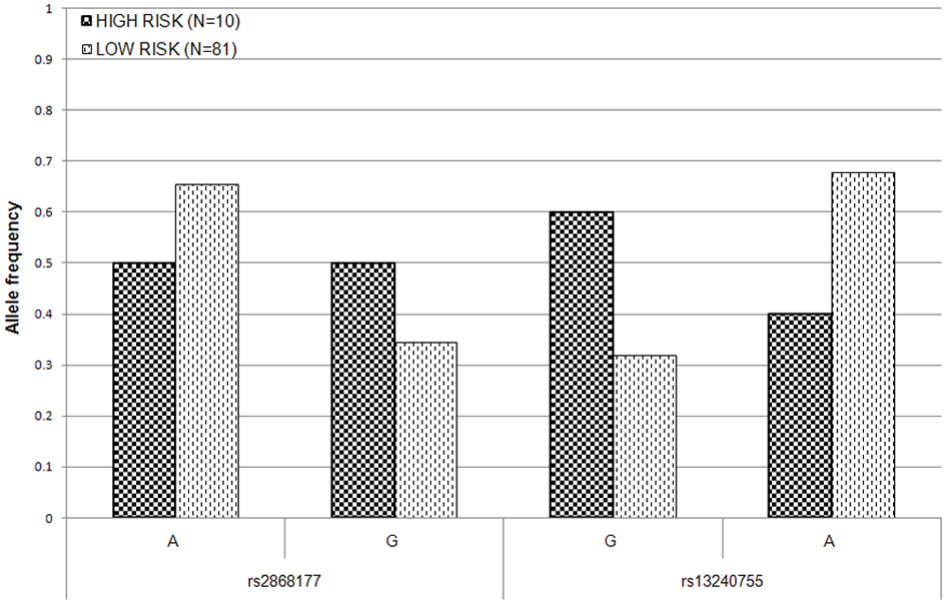
**Allele frequency distribution for the two *POR* SNPs associated with LVEF drop in high- and low- risk groups**. Low risk group consists of patients with LVEF drop of less than 15% after daunorubicin therapy. High risk group consists of patients with at least 15% drop in LVEF after daunorubicin therapy.

To estimate the effect of the two *POR* SNPs on the risk of AIC in *post-hoc* analysis, the patient population was split into high–risk (at least 15% drop in LVEF) and low–risk (less than 15% drop in LVEF) groups. The allele frequency distributions at both loci were significantly different between the two risk groups at 5% level (Figure 3). The difference in allele frequency was especially pronounced at rs13240755, where the minor allele (G) was almost twice as frequent in the high risk (0.60) as compared to low risk group (0.32). For rs2868177, the frequency of the minor allele (G) was also higher in the high risk group (0.50) than it was in low risk group (0.35); however the difference was not as large as for the other SNP. The unadjusted odds ratios (ORs) for rs2868177 and rs13240755 were estimated to be 1.89 (95% CI: 0.7435–4.819; *p* = 0.1756) and 3.18 (95% CI: 1.223–8.27; *p* = 0.01376), respectively.

We caution that these *post-hoc* estimates of effect sizes, variability proportions and ORs do not adjust for the multiple testing and so are descriptive only.

## Discussion and conclusions

The use of anthracyclines at optimal efficacy is hampered by their cumulative dose-dependent adverse side effects, the most serious of which is the AIC that with time can result in anthracycline associated cardiomyopathy (AAC) leading to congestive heart failure. Each anthracycline treatment in addition to killing cancer cells also kills cardiomyocytes, resulting in AIC (De Angelis et al., 2010; Sawyer et al., 2010). Due to the limited regenerative capacity of the heart, cumulative toxicity eventually surpasses a threshold of damage. This triggers a more generic process of ventricular remodeling which leads to AAC (Sawyer et al., 2010). Therefore, careful monitoring and reduction of the risk of AIC during treatment will minimize the threat of developing AAC, which can occur many months or even years later. Biomarkers of the risk of acute AIC could thus greatly aid in optimizing treatment strategies before treatment begins. The relatively high heritability of sensitivity of normal cells to anthracyclines indicates that the inter-patient variability in risk of AIC should at least partially be explained by common genetic variants (Duan et al., 2007; Peters et al., 2011).

The exact mechanism of AIC is still unclear; however experimental evidence suggests that accumulation of toxic metabolites generated during the drug's metabolism significantly contributes to the observed cardiotoxicity (Menna et al., 2012; Visscher et al., 2012). Therefore, we tested SNPs in 60 genes coding for proteins involved in drug metabolism and efflux (Supplemental Table1) for association with AIC in a sample of AML patients undergoing daunorubicin based chemotherapy.

Using the standard single SNP based association test, none of the analyzed SNPs was found to be significantly associated with AIC. However, given that the cardiotoxicity due to anthracycline treatment is a complex, polygenic phenotype it is expected to be weakly associated with single underlying genetic contributors. Such weak associations are missed by the typical “single- SNP-at-a-time” analysis due to the high cost of adjustment for multiple comparisons that are required to control the false discovery rates. In fact, in exploratory studies of multifactorial phenotypes, correction for multiple comparisons has been criticized as an overly stringent requirement demanding unrealistic sample sizes (Miaskowski et al., 2013). On the other hand, as these corrections help to limit the chance of false positive results, we believe that they should be performed, but with relaxed significance thresholds, particularly for complex traits involving a large number of contributing factors of small effect. Indeed, it has been shown that a set of SNPs with significance value (*P* < 0.5) much below the accepted GWAS threshold (P < 5 × 10^−8^) was a better predictor of a complex outcome than a single SNP with *P* < 5 × 10^−8^, despite the fact that many of the SNPs in the set were probably false positive associations (Purcell et al., 2009). This suggests that both the number of undetected (weak) variants as well as their joint effect on the outcome is substantial (Amin et al., 2009).

To minimize the number of missed associations, we used a multi-SNP analysis to identify genes associated with the percent drop in LVEF due to DAUN treatment. This analysis identified P450 oxidoreductase (*POR, CPR, CYPOR, P450R*) as the gene most strongly associated with DAUN induced LVEF drop (FDR-adjusted *p*-value 15%). The human *POR* gene has 16 exons and exons 2–16 code for the 677 amino acid POR protein (NCBI NP_000932.2). The single copy of the 50 kb *POR* gene is located on chromosome 7 (7q11.23). Interestingly, this chromosome was recently found to harbor a region with significant linkage to daunorubicin cytotoxicity (Peters et al., 2011). The *POR* gene encodes a flavoprotein that transfers electrons from NADPH to various proteins, including the cytochrome P450 enzymes. The anthracyclines, as well as other quinone containing compounds, are converted to semiquinone radical forms by microsomes through a one electron reduction catalyzed by POR (Bachur et al., 1979). This bioactivation step stabilizes cross-linking of the drugs with DNA which is thought to significantly increase their cytotoxicity (Kostrzewa-Nowak et al., 2005). At the same time, the anthracycline bioactivation step generates free radicals that may further contribute to more widespread anthracycline induced cellular damage (Gille and Nohl, 1997). There is also evidence that CYP-mediated oxidative metabolism of anthracyclines may contribute to the drug induced cardiotoxicity through generation of oxidative stress and/or disruption of arachidonic acid metabolism (Zordoky et al., 2010).

The *POR* gene is highly polymorphic and a number of *POR* genetic variants were shown to have substrate dependent effects on cytochrome P450 mediated drug metabolism (Gomes et al., 2009; Miller et al., 2011). The three SNPs found here to drive the *POR* association with DAUN induced drop in LVEF are all located in the intronic region of the gene and thus have not been assessed for their effect on POR enzyme function. Interestingly, the SNP rs2868177 has been found to be associated with inter-individual variability in the levels of warfarin maintenance dose, suggesting that it may affect POR mediated metabolism in general (Zhang et al., 2011). The mechanism underlying this association is beyond the scope of this study. We can speculate that these intronic SNPs could be tagging rare, unidentified mutations that affect the protein sequence. Alternatively, as has been shown for other risk associated SNPs in non-coding regions, they could themselves disturb elements that regulate gene expression or features that modify processing (Jensen et al., 2009; Ott et al., 2009; Cowper-Sal Lari et al., 2012). Whichever the case may be, the association of such SNPs with disease risk is not that unusual. In fact, over 70% of risk associated SNPs in the National Genome Research Institute GWAS do not disturb the protein coding sequence (Abecasis et al., 2010).

In *post-hoc* analyses of our study sample, *POR* is estimated to account for some 11.6% of the variability in the drop in LVEF after DAUN treatment, over and above the estimated 13.2% accounted for by cumulative dose and ethnicity. Given that cumulative anthracycline dose is virtually the only factor used clinically to predict the risk of developing cardiotoxicity as a result of anthracycline treatment, the contribution that genetic analyses of *POR* can make to the assessment of this risk is worthy of follow up. The SNP rs13240755 was the most significant driver of the *POR* association with AIC, with individuals carrying the G allele having increased risk of cardiotoxicity (unadjusted OR = 3.18; 95% CI: 1.223–8.27; *p* = 0.01376). These point and interval estimates of the OR are expected to be biased away from one as a result of the multiple genes queried (Ioannidis, 2008). Thus, validation of these results in an independent study is required. Once validated, the *POR* alleles can be included in a panel with other genetic and non-genetic risk factors, to predict AIC. Given that anthracyclines are used in the treatment of a wide variety of cancers including HER2 positive breast cancer, hematological malignancies, soft tissue sarcomas, and many types of carcinomas (http://www.bccancer.bc.ca/HPI/DrugDatabase/DrugIndexPro/Doxorubicin.htm), such a panel of risk factors should improve the clinical outcome of a substantial number of cancer patients. Additionally, this personalized approach to cancer treatment should reduce the cost of health care by eliminating inappropriate treatments (that is, those cases in which the patient derives no therapeutic benefit) and reducing number of conditions caused by adverse drug reactions.

A limitation of this study is the small sample size. However, unfortunate, this candidly is a reflection of the challenges of pharmacogenomic studies in cancer patient populations undergoing heterogeneous treatments (Wheeler et al., 2012; McLeod, 2013). Even in the case of more common cancers, objective characterization of drug response phenotype is faced with lack of uniform treatments and the tumor molecular heterogeneity. The need for relatively homogeneous patient populations for accurate assessment of the genetic effect on drug response will result in a decrease of the study population size. For very common forms of cancer, relatively large, uniform populations can be potentially recruited through a prospective study; however, for less common cancers such approaches would take many years to complete and are impossible for any single research group. The impracticality of randomized, controlled, prospective trials for clinical implementation and a need for a more efficient approach has been recently discussed in more detail elsewhere (Hattersley and McCarthy, 2005; Wheeler et al., 2012; McLeod, 2013; Miaskowski et al., 2013). We believe that publication of preliminary discoveries, even of marginal statistical significance, can facilitate the translation of potential biomarkers into clinics. Such preliminary candidates can be included by other research groups in validation studies. Furthermore, there may be similar findings discovered at an independent research center that are not being published because of similar strict requirements but in fact represent an independent validation sample, as has been the case with a SNP associated with methotrexate clearance (Trevino et al., 2009; Lopez-Lopez et al., 2011). Although the *POR* SNP association with DAUN induced cardiotoxicity has not been reported by other research groups, the SNP has been found to be associated with levels of warfarin maintenance dose (Zhang et al., 2011), and thus may represent a general marker of drug clearance. This speculation is supported by the essential role of POR for the function of the cytochrome P450 system.

Lastly, our study provides additional support for the recent observations which suggest that identification of a comprehensive list of markers for complex traits requires more than a simple increase in the size of study sample, especially since the required sample size for classic genetic association studies of multifactorial phenotypes is estimated to have unrealistic magnitude (Purcell et al., 2009; Yang et al., 2010; Ober and Vercelli, 2011; Han et al., 2012; Urbach et al., 2012; Wheeler et al., 2012). We strongly support the need for modification of the experimental approach and application of statistical models that take into account the multifactorial (genetic and environmental) character of such traits, including interaction between them. As the cost of generating data plummets there is a continually increasing need for more sophisticated approaches to genome wide analyses. The future of personalized medicine now depends on careful attention to study design, and the development and application of more powerful analytic approaches to the resulting data (Urbach et al., 2012; Wheeler et al., 2012).

## Author contributions

Joanna M. Lubieniecka—study design, genotyping, data analysis, manuscript preparation. Jinko Graham—data analysis, manuscript preparation. Daniel Heffner—clinical data preparation. Randy Mottus—study design, manuscript preparation. Ronald Reid—study design, manuscript preparation. Donna Hogge—study design, clinical data preparation, and analysis. Tom A. Grigliatti—study design, manuscript preparation. Wayne Riggs—study design, manuscript preparation.

### Conflict of interest statement

The authors declare that the research was conducted in the absence of any commercial or financial relationships that could be construed as a potential conflict of interest.
